# Monocytes and Macrophages in Spondyloarthritis: Functional Roles and Effects of Current Therapies

**DOI:** 10.3390/cells11030515

**Published:** 2022-02-02

**Authors:** Sara Martínez-Ramos, Carlos Rafael-Vidal, José M. Pego-Reigosa, Samuel García

**Affiliations:** 1 Rheumatology & Immuno-Mediated Diseases Research Group (IRIDIS), Galicia Sur Health Research Institute (IIS Galicia Sur), SERGAS-UVIGO, 36312 Vigo, Spain; sara.martinez@iisgaliciasur.es (S.M.-R.); carlos.rafael@iisgaliciasur.es (C.R.-V.); jose.maria.pego.reigosa@sergas.es (J.M.P.-R.); 2 Rheumatology Department, University Hospital Complex of Vigo, 36214 Vigo, Spain

**Keywords:** spondyloarthritis, monocytes, macrophages, current therapies

## Abstract

Spondyloarthritis (SpA) is a family of chronic inflammatory diseases, being the most prevalent ankylosing spondylitis (AS) and psoriatic arthritis (PsA). These diseases share genetic, clinical and immunological features, such as the implication of human leukocyte antigen (HLA) class I molecule 27 (HLA-B27), the inflammation of peripheral, spine and sacroiliac joints and the presence of extra-articular manifestations (psoriasis, anterior uveitis, enthesitis and inflammatory bowel disease). Monocytes and macrophages are essential cells of the innate immune system and are the first line of defence against external agents. In rheumatic diseases including SpA, the frequency and phenotypic and functional characteristics of both cell types are deregulated and are involved in the pathogenesis of these diseases. In fact, monocytes and macrophages play key roles in the inflammatory processes characteristics of SpA. The aim of this review is analysing the characteristics and functional roles of monocytes and macrophages in these diseases, as well as the impact of different current therapies on these cell types.

## 1. Introduction

Spondyloarthritis (SpA) are defined as a group of chronic inflammatory diseases that affect mainly the spine and joints, being the sacroiliac joint the most typically involved. These diseases, which affect to approximately 1% of the worldwide population, cause serious disorders, pain and disabilities, leading to significant health and socioeconomic problems. In contrast to other rheumatic diseases such as rheumatoid arthritis (RA) and systemic lupus erythematosus (SLE), the prevalence of spondylarthritis is similar between males and females and the onset of the disease (third-fourth decade) is earlier than other rheumatic joint diseases [1,2,3,4,5].

The diseases that are framed within the SpA are ankylosing spondylitis (AS), undifferentiated spondyloarthritis (USpA), axial spondyloarthritis (axSpA), psoriatic arthritis (PsA), reactive arthritis (ReA), enteropathic arthritis and peripheral SpA, being the most predominant AS and PsA [1,6,7,8]. These diseases can be grouped in axial or peripheral depending on the regions of the body that are affected [9], and they share genetic, clinical and immunological features, including joint inflammation (peripheral and axial skeleton), extra-articular manifestations and the absence of diagnostic autoantibodies [10]. The joint affectation, mainly the sacroiliac joint, is a common sign in SpA, but some particularities can be related to the different diagnosis. In fact, low back pain is typically found in AS and UspA [11]; the conjunctivitis-urethritis-polyarthritis triad characterize ReA [12]; enteropathic arthritis patients present chronic bowel inflammation [13]; axial skeleton (spine and the sacroiliac joints) involvement defines axSpA; psoriasis is the hallmark of PsA [14] and peripheral arthritis with dactylitis is distinctive of both peripheral SpA and PsA [11,15]. Some of the most common extraarticular signs are psoriasis, anterior uveitis, enthesitis and inflammatory bowel disease, in order of frequency. Other comorbidities like osteoporosis, cardiovascular events, depression, hypertension and diabetes are also frequent in SpA patients [16,17,18,19]. It is also important to emphasize that, compared with the general population, PsA patients have higher mortality rates, being tumours, vascular disease and respiratory disorders the major causes of death [19].

Cells of the innate immune system represent the first barrier of defence against pathogens. Among these cell types are mucosal-associated invariant T (MAIT) cells, invariant natural killer T (iNKT) cells, gamma delta T cells (γδ T cells), innate lymphoid cells (ILCs), neutrophils, mast cells, eosinophils and monocytes and macrophages [20]. Monocytes and macrophages have an essential role in the activation of innate immune system and after recognition of pathogens release inflammatory cytokines such as TNF, IL-6, IL-1β and chemokines that activate and attract other immune cells to the inflammation sites. In addition, macrophages play an essential role in the defence against bacteria and external agents to degrade them to peptides, performing antigenic presentation at MHC I and II [21]. In addition, these cell types also play important roles in tissue repair and angiogenesis. Rheumatic diseases such as RA, SLE and SpA are characterized by an aberrant activation of immune cells, both from innate and adaptive system [21,22,23,24]. Given the relevance of monocytes and macrophages in the pathogenesis of SpA, this review aims to analyse the role and function of these cell types in these diseases, as well as the impact of different clinical treatments on monocyte and macrophage activity.

## 2. Genetic Factors

Genetic relationship has been established in the pathogenesis of SpA. The strongest association was found in the *HLA-B27* gene, which encodes the human leukocyte antigen (HLA) class I molecule 27 (HLA-B27), which is the major risk factor for the development of SpA, especially AS and USpA [7,25]. Human leukocyte antigens (HLA), also known as Major Histocompatibility Complex (MHC), are responsible for the presentation of intracellular and extracellular peptides to immune system cells for subsequent activation. The HLA-B27 molecule belongs to MHC class I molecules, which present intracellular peptides to CD8^+^ T cells [26]. In addition, several HLA-B27 subtypes are identified with SpA disease phenotypes [27]. For example, HLA–B27 overexpression and the predominance of its subtypes B*2702, B*2705, and B*2707 are described in AS [28]. Remarkably, the presence of *HLA-B27* gene is usually employed as a biomarker for early detection [29] and SpA patients HLA-B27 positive show more radiological signs of joint damage [26]

Besides that, *HLA-C* gene, which codifies HLA-C glycoprotein, is also involved in SpA genetics. HLA-C promotes, among others, the production of cytokines, osteoclast differentiation and monocyte activation. Additionally, single nucleotide polymorphisms (SNPs), such as rs10484554, rs13191343 or rs12191877, and risk alleles in *HLA-C* have been associated to PsA and its variable clinical manifestations. Higher levels of HLA-C protein are related to risk of suffering inflammatory bowel disease (IBD) [30,31,32,33]. 

Other genetic associations have been found between non-MHC genes and SpA, such as the endoplasmic reticulum aminopeptidase 1 gene (*ERAP1*), IL23 receptor (*IL23R*) and *STAT3*, which has been associated with AS by genome wide association studies (GWAS) [34]. *ERAP1* polymorphisms could determine ERAP-1 enzymatic activity, thereby modifying HLA–B27 peptidome and regulating its implications in SpA [35]. Also, genes that encodes for pro-inflammatory genes such as *IL12* and *IL23A* have been linked to PsA [36,37]. 

## 3. Epigenetics and Environmental Factors

Several epigenetic disbalances have been also associated to SpA pathogenesis. Methylation is one of the major epigenetic mechanisms involved in a wide range of diseases, including SpA. Aberrant methylation is consequence of an increased addition of methyl groups in certain gene regions. Thus, hypermethylation of promoter regions induces a decreased expression of genes, usually enhancing pathologic phenotypes [27]. GWAS studies have found differentially methylated positions in the *HLA-DQB1* gene in AS patients. In addition, several hypermethylated genes have been described in SpA with respect to controls. Among them, hypermethylation of *BCL11B*, *IRF8* and DNA methyltransferase genes such as *DNMT1* was found in patients with AS with respect to healthy controls [38,39,40].

MicroRNAs (miRNAs) are also important epigenetic factors and multiple studies have reported dysregulation of different miRNAs in SpA patients. One of the most important works has found a signature of 13 miRNAs deregulated in monocytes, as well as 11 miRNAs deregulated in CD4^+^ T cells, in patients with axSpA compared to controls, which are implicated in the pathogenesis of SpA [41]. In this regard, correlations have been described between some miRNAs and certain clinical parameters of these diseases. For example, differential expression of miR-146a-5p, miR-125a-5p and miR-22-3p has been correlated in serum samples from SpA patients with TNF and C-reactive protein levels [42].

In addition, other related epigenetic marks have been reported in SpA. This is the case of histone modifications, as AS patients treated with a TNF inhibitor showed increased activity of the enzyme histone acetyl transferase, indicating increased acetylation and thus increased gene expression in these acetylated regions [43]. Also, increased levels of Histone Deacetylase 3 (HDAC3) were found in the peripheral blood of AS patients [44,45].

Environmental factors are also involved in SpA and, for instance, previous infections have been associated with the development of some SpA. A link has been found between the gut flora microbiota and inflammation in these diseases as well [7,46,47]. In fact, a rat model of SpA has shown that intestinal inflammation, dependent of microbiota, enhances bone erosive potential in monocytes. Besides, bowel disruption promoted systemic inflammation, osteoclastogenesis and joint destruction [48]. Conversely, in vitro studies revealed that anti-IL-17 drugs contribute to dysbiosis and gut inflammation through inhibition of IL-17 pathway [49,50]. Finally, mechanical stress seems to be a determining factor in the appearance and development of SpA [51].

## 4. Etiopathogenesis

Despite the numerous studies in the field, the molecular mechanisms of action involved in the pathogenesis of SpA are still unclear [52]. There are currently different hypotheses, which can occur in combination and have in common that the trigger for autoinflammatory processes are mechanisms mediated by the HLA-B27 antigen. First hypothesis postulates that certain HLA-27 subclasses bind to peptides that are recognized by CD8^+^ cells, which leads to the activation of autoreactive T cells [46,53]. The second hypothesis suggests that defective HLA-B27 folding, occurring at the endoplasmic reticulum of immune cells, trigger the activation of the unfolding protein response (UPR) pathway, which induces the translocation and therefore activation of transcription factor NF-kB to the nucleus, leading to the production of cytokines involved in the pathogenesis of the disease by different inflammatory cells [46]. Related to this, it has been recently seen in macrophages that HLA–B27 impaired ubiquitination might promote the accumulation of misfolded HLA–B27 dimers, which are involved in the pathogenesis of SpA [54]. Moreover, murine models have revealed SpA-associated unconventional HLA-B27 molecules, detected in monocytes, resident and infiltrating macrophages [55]. The third hypothesis postulates that, since HLA-B27 tends to form homodimers, these are recognized as self-antigens by T cells and natural killer cells, leading to increase the production of cytokines such as IL-17 and IL-23 [46,52,56]. Also, a fourth hypothesis indicates that different polymorphisms in the aminopeptidases *ERAP1* and *ERAP2* genes would be involved in the production of aberrant forms of HLA-B27 and in the modification of peptides bound to HLA-B27. Finally, the most recent hypothesis links gut inflammation and dysbiosis with HLA-B27 and susceptibility to the development of SpA [52].

Overall, the mechanisms of action of these diseases involve the production of autoreactive T cells, through the activation of CD8^+^ T cells and through a key role of antigen presenting cells such as dendritic cells and macrophages. Following this activation, CD8+ T cells activate and perpetuate the inflammatory process, mainly through the release of key cytokines such as IL-17, IL-23, TNF, and IL-6; chemokines and other mediators such as Receptor Activator of Nuclear Factor-κB (RANK). The final consequences of these processes are the inflammation of the affected tissues (joints, skin, eyes, gut) and the destruction of the joints and spine, due to impaired osteoclastogenesis and bone remodelling [57].

## 5. Cell Pathology: Monocytes, Macrophages and Osteoclasts in Spondyloarthritis

### 5.1. Monocytes

Cells of the innate immune system are involved in the onset and development of SpA and, within this group, monocytes have been shown to play a key role in the pathogenesis of these diseases [58]. According with the established literature, there are three classes of monocytes, defined by the expression of the surface markers CD14 and CD16. The classical monocytes, which are the most abundant population (around 90%), show high CD14 expression but no CD16 (CD14^++^ CD16^−^), meanwhile the non-classical express a low level of CD14 together with high CD16 (CD14^+^CD16^++^). Finally, the intermediate monocytes present high levels of CD14 and low CD16 expression (CD14^++^CD16^+^) and are considered a transitional population between the classical and non-classical monocyte subsets [58,59]. However, these 3 populations of monocytes reveal unique characteristics in healthy individuals, as they are defined by different transcriptional profiles, possess different repertoires of cell surface receptor genes and distinct cytokine production patterns revealed by LPS activation [59].

Similarly to other diseases, including RA, there is a disbalance in the frequency of three monocyte subsets in SpA patients [60]. In contrast to RA patients, who showed an increase of intermediated monocytes [61], an increment in the frequency of classical monocytes has been observed in SpA [62]. In addition, another study found an expansion of CD14^++^ CD16^+^ CCR9^+^ CX_3_CR1^+^ CD59^+^ monocytes in the peripheral blood, synovial fluid, synovial tissue and bone marrow of AS patients. Importantly, this population positively correlated with disease activity parameters and levels of C-reactive protein, and these cells displayed more pronounced phagocytic activity in AS patients than in controls [63]. Besides the frequency of monocyte subset, the phenotypic characteristics of monocytes are also altered in SpA patients. For instance, monocytes from AS patients exhibit higher pro-inflammatory phenotypes secrete higher amounts of pro-inflammatory cytokines and proteomic analysis showed a higher activation of leucocyte extravasation vascular, endothelial growth factor, Janus kinase/signal transducer and activator of protein (JAK/STAT) and TLR pathways [64,65]. Related to this, the levels of TLR4 were higher in monocytes from SpA and PsA patients compared to healthy controls [66]. Moreover, monocytes from PsA patients show higher expression of the calcium-binding proteins S100A8/A9, which have a central role in controlling leukocyte trafficking and the metabolic processes of arachidonic acid [67].

On another note, the monocytes to lymphocytes ratio (MLR) was increased in patients with AS compared with non-radiographic axSpA, another of the findings that highlights the role of monocytes in SpA. Furthermore, this ratio was correlated with C-reactive protein (CRP) levels, erythrocyte sedimentation rate (ESR) levels and spine movements [68].

Lastly, it is important to highlight that the monocytes are of great importance in these diseases because they are precursors of two key cell types in SpA: macrophages and osteoclasts, as we will discuss in the next sections [69].

### 5.2. Macrophages

Macrophages are other cells belonging to innate immune system that are present in all tissues and body compartments and serve as the first line of defence against infection. They are the main phagocytic cells, but they are also antigen presenters and secrete cytokines involved in the immune system activation. Macrophages play also key roles in the maintenance of tissue homeostasis and are critical cells in the orchestration of chronic inflammation observed in different diseases, including SpA [24,60,70,71]. Regarding the ontology of this cell type, it was accepted during decades that all macrophages originate from circulating adult blood monocytes. However, works from last years have shown that tissue macrophages, including microglia, Kupffer cells, Langerhans cells and kidney, alveolar, heart and synovial macrophages, originate during embryonic development from the yolk sac, fetal liver or hematopoietic stem cells [72,73].

The phenotypic characteristics and functional capacities of macrophages are defined by the environmental factors, such as cytokines and pathogens that they are exposed during the differentiation process. Historically, macrophages have been classified into classically activated pro-inflammatory M1 or alternatively activated M2 wound-healing and immunosuppressive macrophages [71]. M1 macrophages, which are induced by IFN-γ, LPS and GM-CSF, are key components of host defence and are characterized by the expression of the pro-inflammatory cytokines IL-1β, IL-6, IL-12, IL-23 and TNF. The Th2 cytokines IL-4 and IL-13 induce the differentiation of wound-healing macrophages, which secrete components of the extracellular matrix and are involved in the tissue homeostasis. Finally, IL-10 drives immunosuppressive M2 macrophages that dampen the immune response and limit inflammation, playing this manner a regulatory role [21,70,71]. In addition to the different functional characteristics, M1 and M2 macrophages also express specific surface markers, such as CD80 and CD64 (M1) and CD200R and CD163 (M2) [74]. This is a useful classification for the in vitro differentiation of macrophages; however in vivo distinction is not easy, as macrophages can show characteristic of both M1 and M2 macrophages and they show a broad heterogeneity. In fact, recent studies have reported the existence of several macrophage subsets in the synovium of RA patients [21,75,76].

Phenotypic characterization of the SpA lining and sublining layers has shown an increased expression of the M2 macrophage marker CD163 compared to RA synovium [77,78]. Importantly, CD163 was also increased in the colonic mucosa of SpA and Crohn’s patients versus ulcerative colitis patients and healthy controls [78,79]. The expression of M1 macrophages is more controversial, as an initial work found a reduction of CD80 and CD86 expression in the synovium of SpA compared to RA patients [78], but further works have not validated this finding, neither difference in the expression of CD14, CD68 CD64 or CD200R [77,80]. In addition, CD163 synovial expression was correlated with clinical disease parameters, such as swollen joint count (SJC), serum CRP and ESR [78]. In vitro studies also support these findings, as the expression of the M2 markers CD163 and CD200R is induced in peripheral blood monocytes by the stimulation with the synovial fluid of SpA, but not RA patients. In addition, the synovial fluid of SpA patients showed a reduced expression of TNF, IL-1 and CXCL-10, mediators secreted by M1 macrophages [81]. Altogether, these data suggest that in SpA M2 macrophages predominate over M1 phenotype. These M2-like phenotypic characteristics may be responsible of the differences in the synovium of SpA and RA patients, such as the reduced infiltration of B and T cells, higher frequency of Th17 cells and the increased vascularity, with more tortuous vessels found in SpA patients [78,82].

However, despite the M2-like phenotype of SpA macrophages, these cells are able to produce inflammatory cytokines. CD163^+^ SpA macrophages express high levels of HLA-DR and secrete TNF, but not IL-10 [78]. Moreover, TNF induced the expression of inflammatory mediators by monocyte-derived macrophages from blood and synovial fluid (SF) of PsA patients, as well from monocytes of healthy controls differentiated with the SF of PsA patients. Interestingly, activation of Tie2 signalling enhanced the TNF-dependent expression of these inflammatory mediators [83,84]. As Tie2 is also involved in angiogenesis and Angiopoietin-2, one of the Tie2 receptors, is elevated in the synovium of SpA patients, macrophage Tie2 signalling may be essential for the pathogenesis of SpA [85,86].

Importantly, IL-17, a key cytokine in SpA pathogenesis, stimulates the production and expression of proinflammatory cytokines by human macrophages [87]. Macrophages also express mediators that are elevated in SpA and are involved in the perpetuation of inflammation, such as Ca^2+^ binding proteins S100A8 and S100A9 and HMGB1 (high mobility group box 1 proteins) [88,89], and angiogenic factors (vascular endothelial growth factor –VEGF- and basic fibroblast growth factor –BFGF-) that are highly expressed in the synovium of early PsA patients [85]. Finally, macrophages also contribute to the joint destruction through the production of matrix metalloproteinases, including MMP-2, MMP-3, MMP-7 and MMP-9 [90,91].

### 5.3. Osteoclasts

Homeostatic bone tissue remodelling is consequence of a tight balance between the levels of bone formation (mediated by osteoblasts) and bone resorption (induced by osteoclasts). In SpA there is an imbalance in bone tissue remodelling, which leads to bone destruction and resorption, mainly at the peripheral joints, but also to new bone formation in the spine triggering disk fusion. 

Osteoblasts have a mesenchymal origin, meanwhile osteoclasts are multinucleated cells of hematopoietic origin. Osteoclast development is controlled by the interaction of TNF superfamily receptor-ligand pair known as Receptor Activator of Nuclear Factor-κB (RANK) and RANK ligand (RANKL), which are both necessary and sufficient requirement for osteoclast formation [92]. RANKL is over-expressed in SpA cells, notably in macrophages and memory T lymphocytes, but also in synovial fibroblasts and osteoblasts [57]. This enhanced expression of RANKL is mediated by different cytokines, such as TNF, IL-17, TGF-β and IL-22 [93,94]. Besides the IL-17 mediated RANKL expression, IL-17 also plays a direct role in bone resorption through the expression of RANK in osteoclast precursors [93,95]. The role of IL-23 on osteoclastogenesis seems more controversial and different studies have shown that its implication in this process is due to the induction of Th17 cells and Il-17 secretion, rather than a direct effect [95]. Interestingly, sera from patients with axSpA, which showed higher levels of TNF and IL-17 compared to healthy control, promoted the expression of RANK during the osteoclastogenesis process, highlighting the prominent role of both cytokines [96]. 

Other mediators have been involved in bone remodelling. For example, Macrophage-colony stimulating factor (M-CSF) and IL-34, which maintain macrophages homeostasis and regulates osteoclasts, are 1raised in PsA serum and these levels are associated with bone erosion [97]. In addition, inhibition of the share receptor (CSF-R1), reduced the severity of arthritis and the bone destruction in an arthritis mouse model [98].

## 6. SpA Treatments and Effect on Monocyte/Macrophage Function

The European Alliance of Associations for Rheumatology (EULAR) and the American College of Rheumatology (ACR) have recently stablished medication guidelines for treating Spondyloarthritis. To date, the first line of pharmacological treatment of axSpA is the non-steroidal anti-inflammatory drugs (NSAIDs) therapy, followed by glucocorticoids, while second and third lines correspond to Biological disease-modifying antirheumatic drugs (DMARDs), relegating Non-Biological DMARDs to the last option. According to guidelines, Biologicals DMARDs employed are TNF inhibitors, IL-17 inhibitors and JAK inhibitors, in order of preference. In PsA, the use of Non-Biological DMARDs prevails over NSAIDs and the administration of IL-12/23 inhibitors is suggested [99,100,101,102]. Nevertheless, new-targeted medications are being studied. Below these lines, we analyse their effects on monocytes and macrophages populations. Also, the mechanism of action and effects of treatments in monocytes and macrophages are summarized in Table 1.

### 6.1. NSAIDs

Despite non-steroidal anti-inflammatory drugs (NSAIDs) are the first-line drug treatment for SpA, not much evidence is found about the effect of these drugs in SpA monocytes/macrophages. NSAIDs inhibit the activity of cyclooxygenase (COX), which modulates prostaglandin E2 (PGE_2_) expression. PGE_2_ is an important early mediator of enthesitis, the hallmark of SpA, and is involved in the differentiation of Th17 cells [116], and, therefore, could activate monocytes/macrophages in an indirect manner. In addition, previous studies showed an immune modulatory role of NSAIDs, as these drugs attenuated inflammatory processes induced by macrophages and T cells, both key cell types in the pathogenesis of SpA [103].

However, a study in axSpA patients found that NSAID treatment did not modulate the monocytes secretion of IL-1β, IL-6, and TNF, in contrast with the effect of inhibitors (TNFi). These results suggest that NSAIDs are not important modulators of monocytes/macrophages function [65]. In contrast to this lack of action on dampening inflammation, NSAIDs minimize radiographic signs of spinal damage progression in axSpA [117,118] and this effect might be due to the reduced COX/PGE_2_ activity, which would reduce Th17 differentiation, leading to decreased osteoclastogenesis. Nevertheless, further studies are needed for validating this hypothesis.

### 6.2. Glucocorticoids

The administration of glucocorticoids is another line of treatment for SpA patients, but the utilization is controversial because of their adverse effects, as osteoporosis and increase of cardiovascular risk. However, a recent systematic review has demonstrated the efficacy of using glucocorticoids in the short term (≤6 months) in SpA. Importantly, no deaths or major adverse events were reported [119]. Glucocorticoids signal through the glucocorticoid receptor (GCR), which activate cellular pathways that modulate the activity of the transcription factors C/EBPβ, PPARs and NFκB. These transcription factors promote the expression of anti-inflammatory mediators [120] and inhibit the expression of inflammatory mediators, including prostaglandins (departing from COX2 transcription blocking) [119,121] and Phospholipase A2 in the arachidonic acid pathway [122]. The overall consequence is the resolution of the inflammation [123,124,125].

In monocytes, glucocorticoids increase IL-10 secretion [126] and deplete the proinflammatory CD16^+^ cells [104]. It has been proven that glucocorticoids also mitigate some of the effects of macrophage activation, such as the production of important macrophage mediators, including TNF and IL-6. However, macrophages produce large amounts of pro-inflammatory cytokines, and doses of corticosteroids that are potentially toxic if sustained for more than few days are needed for reducing this production [127]. TNF also takes part in intervertebral disc degeneration, suggesting that corticosteroids could reduce pain and, remarkably, the damage in intervertebral discs via reducing monocyte/macrophage TNF secretion [128]. Finally, it has recently been reported that glucocorticoids may act on macrophages inducing a phenotype involved in tissue repair, but this effect needs to be demosntrated in SpA pathogenesis [120].

### 6.3. Non-Biological Disease-Modifying Anti-Rheumatic Drugs 

Non-Biologicals disease-modifying antirheumatic drugs (DMARDs) are widely used for the treatment of rheumatic diseases, including SpA. Methotrexate (MTX) is the most common DMARD and, due to its effect modulating cell-specific signalling pathways, inhibits important pro-inflammatory properties of different cell types involved in the pathogenesis of rheumatic diseases, including T cells, macrophages, endothelial cells and fibroblast-like synoviocytes [105,129]. In the context of this review, MTX induces apoptosis of monocytes and reduces the expression of IL-1β by monocyte precursors and the expression of Fcγ receptors on monocytes of RA patients, demonstrating an anti-inflammatory role [105,106].

In addition, MTX induces release of adenosine by different cell types and the binding of adenosine to the adenosine receptor (ADORA) 2_a_ and 3 reduces the monocyte secretion of TNF and IL-6, and induces the polarization of macrophages towards an anti-inflammatory M2 phenotype. These data suggest an anti-inflammatory effect of MTX on monocyte/macrophages in an indirect manner [129,130]. However, another study showed that MTX dose-dependently induced the expression of IL-1, IL-6 and TNF in a monocytic cell line, which could be implicated in the adverse effect of MTX use in some rheumatic patients [131]. Finally, a study in 10 PsA patients showed that MTX treatment reduced the number of CD68^+^ macrophages in the synovial tissue, as well the expression of inflammatory mediators secreted by monocytes/macrophages, such as IL-1β, IL-8, TNF and MMP-3 [132].

### 6.4. Anti-TNF Treatments

TNF is one of the key cytokines in the pathogenesis of SpA and is involved in different pathogenic processes of the disease, like inflammation, angiogenesis and osteoclastogenesis. TNF signals through TNF receptors (TNFR), mainly TNFR1 and TNFR2. After receptor binding, TNF activates two different pathways. On one hand, TNF activates MAP kinase pathways, such as c-Jun N-terminal kinases (JNKs), which induces the translocation of the transcription factor AP-1 to the nucleus. On the other hand, TNF activates another signalling pathway leading to the phosphorylation and degradation of IκBα, which promote the nuclear translocation of NF-κB. Both transcription factors ultimately trigger the expression of pro-inflammatory, anti-apoptotic, angiogenic and cell proliferation genes, which most of them are involved in the pathogenesis of SpA [133]. For that reason, there are currently several approved anti-TNF drugs for the treatment of these diseases [134]. 

Etanercept was the first anti-TNF therapy approved for therapeutic use in SpA. This drug is a TNF receptor fusion protein and is approved in different spondyloarthropathies: PsA, AS and non-radiographic axSpA. Regarding its function in monocytes and macrophages, recent studies has shown that etanercept skewed macrophage polarization towards a M2 phenotype and that etanercept reduced the expression of LPS-induced expression of NF-κB target genes and the LPS-induced MMP9 activity in SpA monocytes [107,108,109].

Adalimumab and Infliximab are recombinant human monoclonal antibodies that blocks TNF. They are currently approved for the treatment of several SpA, specifically PsA, plaque psoriasis, AS, ulcerative colitis and Crohn’s disease [9,134]. Both TNF inhibitors (TNFi) restrict pathological angiogenesis and inflammatory cell infiltration in the synovium, including macrophage infiltration of the sublining layer [135]. In PsA patients, Adalimumab leads to a decrease in the number of tissue resident macrophages (CD163^+^), infiltrating macrophages (MRP8^+^) and early stage differentiated macrophages (MRP14^+^) [136], while Infliximab significantly reduces the CD68^+^ macrophage levels in synovial tissue of PsA patients [137,138]. Moreover, both TNFi also enhance in vitro nonclassical monocytes, decrease classical monocytes [139] and modulate macrophage polarization to M2 phenotype [109]. In vitro research studies have shown that both Adalimumab and Infliximab inhibit IL-12/IL-23 production in M1 macrophages through the formation of immune complexes, demonstrating a functional effect of these TNFi in these cell types [140]. Finally, Infliximab also inhibits the osteoclast resorptive activity in AS patients [141].

On the other hand, Certolizumab pegol and Golimumab are also two anti-TNF monoclonal antibody-based treatments used and approved for SpA. Previous research has shown that Certolizumab, as well as Infliximab and Adalimumab, induces differentiation of an immunosuppressive macrophage subtype that inhibits T-cell proliferation [142].

### 6.5. Anti-IL-17 Treatments

Since IL-17 is an essential cytokine involved in the pathogenesis of SpA, therapies against this cytokine have been developed in the last years. There are currently two approved anti-IL-17 treatments for SpA: Secukinumab and Ixekizumab. Secukinumab is a treatment whose target is the cytokine IL-17A and its structure is a fully human monoclonal IgG1k antibody. It has been validated in several SpA diseases, such as AS [143] and PsA [144]. Likewise, Ixekizumab is an anti-IL-17A drug and is a humanized IgG4 monoclonal antibody. Its efficacy has been validated in different SpA types, such as PsA [145] or AS [146]. Besides that, there are currently several anti-IL-17 drugs of clinical interest in development, such as Bimekizumab [147] and Afasevikumab [148].

Different macrophage subpopulations express several subunits of the IL-17 receptor (IL-17R). Macrophage IL-17 signalling is mediated through IL-17R and downstream signalling leads to the activation of C/EBP proteins and the nuclear translocation of NF-kB. This results in the release of a group of key cytokines such as IL12p70, GM-CSF, IL-3 and IL-9, representing a specific cytokine response profile [149,150]. Thus, direct blockade of IL-17 by binding to an anti-IL17 antibody prevents the ligation to IL-17R, inhibiting this manner the release of the aforementioned inflammatory mediators and therefore the pathogenic pathways that trigger macrophage activation and stimulation. The functional consequences in the context of SpA would be the abrogation of monocyte/macrophage activation and the reduction of IL-17-mediated osteoclastogenesis [110,111]. In fact, recent studies reveal that anti-IL17 drugs, in particular Secukinumab, reduce macrophage infiltration and MMP-3 expression, besides controlling disease signs, all without compromising systemic immune response [112].

### 6.6. Anti-IL-12/Anti-IL-23 Therapy

IL-12 and IL-23 are cytokines involved in the pathogenesis of autoimmune and immune-mediated diseases. Both cytokines present the p40 subunit and play a key role in immune cell regulation. IL-12 primarily mediates Th1 responses, enhancing IFN-γ production by NK cells and T cells, leading ultimately to the skewing towards M1 macrophages. On the other hand, IL-23 is crucial for the Th17 differentiation and is involved in the production of IL-17A and IL-17F by NK cells. In terms of signalling mechanisms, both IL-12 and IL-23 share similar pathways, such as JAK2, TYK2, STAT1, STAT3, STAT4 and STAT5 [151,152].

Ustekinumab is a monoclonal antibody against IL-12 and IL-23 approved in ulcerative colitis, Crohn’s disease and PsA. Its mechanism of action consists in the binding of the antibody to the p40 subunit present in both IL-12 and IL-23, inhibiting both cytokines. Inhibition of this subunit is effective in SpA because the IL-23/IL-17 axis plays a key role in the pathogenesis of these diseases, and macrophages are involved in this process, as they are the main producers of this cytokine. Thus, on one hand, IL-23 blockade inhibits Th17 differentiation and a decrease in IL-17 levels. And, on the other hand, the blockade of IL-12 impairs Th1-lymphocytes differentiation [153]. 

In addition, a recent functional study of the effect of Ustekinumab on PsA has yielded important results. In this study, a significantly lower infiltration of CD68^+^ macrophages in the synovial sublining layer was found, which ameliorates the pathogenesis of the disease. Regarding expression of inflammatory mediators, Ustekinumab reduced the levels of IL-23p19 and MMP3 the in synovial tissue biopsies of these patients [113].

### 6.7. JAK Inhibitors

Janus kinase (JAK)/STAT pathways are essential in the inflammatory processes observed in rheumatic diseases [154]. There are different JAK family members (JAK1-JAK3 and TYK2), being JAK1 the most important. In the context of monocyte/macrophages, JAK/STAT pathways are involved in different processes. On one hand, JAK/STAT signals the response to cytokines involved in macrophages differentiation [155]. For example, the binding of IFN-γ to its receptor activates JAK/STAT pathways, leading to M1 macrophages differentiation. On the other hand, IFN-γ, alone or in combination with IL-12, signal through JAK2-Tyrosine kinase 2 (TYK2) or JAK1-JAK2 pathways and induce the release of TNF by macrophages, which contributes to the development of the disease [154].

Due to the prominent role of JAK/STAT pathways in the production of inflammatory mediators, JAK inhibitors (JAKi) have emerged in the last years as therapeutic options of great relevance for the treatment of several immune-mediated inflammatory diseases. JAKi reduce the production of IL-12 and IFN-γ, which promote a decrease in the levels of TNF. Besides that, JAK inhibition also can cause the reduction of other cytokine production directly or indirectly, such as IL-17, IL-23, IL-18, IL-1, IL-6, IL-7, IFN-α and IFN-β, improving the inflammatory status [114]. However, and despite multiple inflammatory cytokines signal through JAK/STAT, the JAKi do not appear to have a direct effect on cytokine targets such as TNF or IL-17. The mechanism of action of JAKi in rheumatic diseases might be the interaction with alternative cytokine pathways [154]. 

Since JAK receptors are also involved in different proinflammatory signalling pathways observed in the pathogenesis of SpA, JAKi are promising therapeutic options [156]. In fact, Tofacitinib, a JAK1 and JAK3 inhibitor, is a drug already approved in PsA [157] and with promising results in a phase III trial in AS [158]. However, the effect of Tofacitinib on SpA monocyte/macrophages activation is still unknown and further research is needed in this context.

### 6.8. Other Biological Therapies

#### 6.8.1. CTLA4-Ig (Abatacept)

Abatacept, an immunoglobulin against Cytotoxic T-Lymphocyte Antigen 4 (CTLA4-Ig), is a selective modulator of T cells employed in SpA treatment [159]. Abatacept inhibits CD4^+^ T cell activation, but also it has been observed that Abatacept reduces the migratory capacity of monocytes from RA patients [115,160]. Moreover, Abatacept downregulates the macrophages TNF production induced by activated T cells [161] and a recent paper has shown that Abatacept is able to directly skew RA macrophages towards a M2 phenotype [162].

#### 6.8.2. IL-6 Inhibitors

Despite the implications of IL-6 in SpA pathogenesis, its inhibition has completely failed [163,164], except in anti-TNF-refractory aggressive SpA [165]. The treatment weakness could happen, according to mouse model experiments, because the pathological mechanisms in tissue resident cells of only few SpA patients would be dependent of IL-6 [166].

### 6.9. Directed Therapies: From Monocytes and Macrophages to Disease Management

Directly targeting monocytes and macrophages or their pathways is a potential novel therapeutic in SpA, as they are both implicated in the pathophysiology of the diseases. Therefore, they are promising therapeutic approaches, but they are still not approved for the clinical use.

#### 6.9.1. Granulocyte–Monocyte Colony Stimulating Factor (GM-CSF) Inhibition

In chronic inflammation, hematopoietic stem cells are redirected to myelopoiesis for granulocyte-monocyte progenitors (GMPs) formation. Particularly, in SpA, these GMPs gather in bone marrow, spleen and affected joints, contributing to disease progression. Furthermore, secreted granulocyte-monocyte colony stimulating factor (GM-CSF) is a cytokine essential for the proliferation and differentiation of myeloid cells, including monocytes. Innate lymphoid cells, IL-17A^+^ T cells and mast cells secrete granulocyte-monocyte colony stimulating factor (GM-CSF). As these cell types are elevated in SpA patients, consequently the levels of this cytokine are also elevated [167,168,169]. Functionally, GM-CSF also induces monocyte inflammatory activity [170]. Taking this into account, antibody blocking of GM-CSF reveals potential therapeutic value in SpA, as it is being tested in other rheumatic diseases [169,171].

In animal models, neutralizing antibodies reduces arthritis signs [172,173]. Some anti-GM- CSF has been already assessed in several rheumatic processes, including SpA, as Mavrilimumab [171,174], Namilumab [175,176] or Otilimab [177], while new molecules are now being tested in randomized controlled trials (ClinicalTrials.gov Identifier: NCT03622658; NCT04205851). 

#### 6.9.2. Apheresis

Granulocyte and monocyte apheresis, an extracorporeal therapy consisting of selective removal of monocytes and macrophages from blood, is a safe procedure that could have an application in SpA, as it is propose for PsA and other rheumatic diseases [178,179]. However, further studies are needed to fully elucidate the therapeutic use in SpA. 

## 7. Concluding Remarks

**Figure 1:**
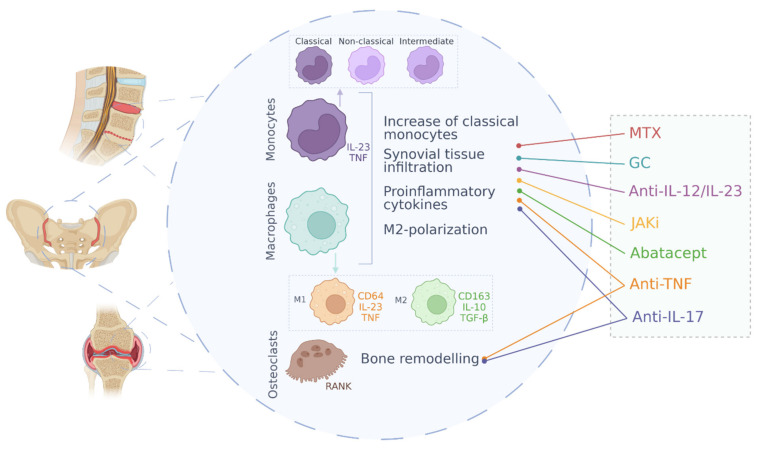
Schematic overview of monocytes and macrophage roles in spondyloarthritis and effect of current therapies. MTX (methrotrexate), GC (glucocorticoids), JAKi (JAK inhibitors).

In this review, we have summarized and analyzed the main molecular mechanisms involved in the pathogenesis of spondyloarthritis and the function of monocytes and macrophages in these diseases (Figure 1). As it has been observed, approved drugs for the treatment of spondyloarthropathies have a clear involvement at the molecular level with the mechanisms of action of monocytes and macrophages in these diseases. This indicates a preponderant role of monocytes and macrophages in SpA and highlights these cell types as a promising target for the development of new therapies.

## Figures and Tables

**Table 1:** Treatments in SpA, mechanisms of action and its effects on monocytes and macrophages.

Treatment Strategy	Drug	Mechanism of Action	Main Effects on Monocytes/Macrophages
NSAIDs	Meloxicam, Ibuprofen, Meclofenamate, etc.	Cyclooxygenase activity inhibition	Decreased activation of macrophages [103]
Glucocorticoids	Prednisolone, methylprednisolone	Phospholipase A2 suppression; NF-κB antagonization	Increased IL-10 secretion by monocytes; CD16+ cells depletion; restrained activity of pro-inflammatory cytokines [104]
Non-biological DMARDs	Methotrexate		Down-regulation of monocytes and macrophages activation; production of cytokine inhibitors by monocytes and IL-1β suppression [105,106]
Anti-TNF	Adalimumab, Infliximab, Certolizumab pegol, Golimumab and Etanercept	TNF antibody blocking and TNF receptor blocking	Decrease of macrophage infiltration in the synovial tissue; decrease of expression levels of metalloproteinases; differentiation of immunosuppressive macrophages [107,108,109]
Anti-IL-17	Secukinumab, Ixekizumab, Bimekizumab and Afasevikumab	IL-17 antibody blocking	Decrease of macrophages infiltration and MMPs expression; reduction of IL-17-mediated osteoclastogenesis [110,111,112]
Anti-IL-12/IL-23	Ustekinumab	Antibody blocking of IL-12 and IL-23 cytokines	Lower infiltration of CD68+ macrophages in the synovial sublining layer [113]
JAK inhibitors	Tofacitinib	JAK1 and JAK3 inhibition	Decreased production of pro-inflammatory cytokines by macrophages [114]
CTLA4-Ig	Abatacept	Immunoglobulin against Cytotoxic T-Lymphocyte Antigen 4	Decrease of TNF production; regulation of migratory capacity [115]

## Data Availability

Not applicable.
